# High-Quality Draft Single-Cell Genome Sequence of the NS5 Marine Group from the Coastal Red Sea

**DOI:** 10.1128/genomeA.00565-18

**Published:** 2018-06-21

**Authors:** David K. Ngugi, Ulrich Stingl

**Affiliations:** a Red Sea Research Center, King Abdullah University of Science and Technology, Thuwal, Saudi Arabia

## Abstract

The uncultured NS5 marine group represents one of the most ubiquitous flavobacterial bacterioplankton associated with marine blooms in the pelagic ocean. Here, we present a single-cell genome sampled from coastal waters in the Red Sea that represents the first high-quality draft genome sequence within the NS5 lineage.

## GENOME ANNOUNCEMENT

The uncultured N5 marine group (also known as ARC11) represents one of the most ubiquitous flavobacterial bacterioplankton in the pelagic ocean (1–3). Their biomass can reach 6 × 10^3^ cells per ml (4), and their abundance is strongly correlated with marine phytoplankton blooms (4–6). Therefore, members of this group are currently considered indicator species for coastal bacterioplankton communities (3). However, to date no member of NS5 has been cultivated or sequenced.

Although the biogeography of this group has been extensively captured in environmental 16S rRNA gene surveys (3–7), information regarding the potential metabolism and ecology of NS5 members in the pelagic ocean currently remains fragmentary. However, environmental fosmids carrying 16S rRNA gene sequences affiliated with NS5 suggest capabilities for degrading high-molecular-weight organic matter such as proteins and polysaccharides (2, 8). Here, we report on a nearly complete single-cell genome (*Bacteroidetes* bacterium SCGC AAA795-G10) of an NS5 member from the coastal waters of the central Red Sea.

Surface seawater samples were collected from Red Sea coastal waters near King Abdullah University of Science and Technology (KAUST) Harbor (22°18ʹ23.20ʹʹN, 39°6ʹ10.71ʹʹE) in February 2012, where the temperature was 25°C and salinity was 39%. Single cells were sorted and amplified using the REPLI-g kit (Qiagen) at the Single Cell Genomics Center in the Bigelow Laboratory for Ocean Sciences as described in Ngugi et al. (9).

A paired-end sequence library (101 bp × 2) of the single-cell amplified genome (SAG) was prepared using the TruSeq DNA library kit and sequenced using an Illumina HiSeq sequencer at the Bioscience Core Laboratory at KAUST. Sixty-five million reads were quality trimmed using Trimmomatic v0.32 (10) and assembled into contigs with SPAdes v3.9.0 (11), applying the error correction and single-cell modes. Genome completeness was estimated using CheckM (12). Carbohydrate active enzymes were predicted using the dbCAN server (13).

The SAG comprises 106 contigs totaling 1.93 Mbp (*N*_50_ value of 1.06 Mbp; 1,900× coverage) with a DNA G+C content of 31.8%. Based on operational standards for SAGs (14), this SAG is of high quality, with 92.1% completeness and 0.5% contamination. It contains 1,746 protein-coding genes and 33 RNA-coding genes, including 1 rRNA operon, as annotated with PGAAP (15) and the RAST server (16). The 16S rRNA gene sequence shows 99% identity to environmental genomic DNA fosmid sequences from the North Atlantic Ocean (2, 8) and the Peruvian coastal margin (GenBank accession number EU795293) and only 95% identity to Arctic fosmids (EU795086 and EU795252). The SAG encodes 37 predicted glycoside hydrolases (GH); among these are endo-β-glucanases (GH16 and GH74), α-fucosidases (GH29 and GH95), α-amylases (GH13), glucosidases (GH3 and GH63), and mannan endo-α-1,2-mannosidase (GH99), indicating the capacity to degrade marine polysaccharides. However, no putative proteorhodopsin-encoding genes were detected, as is the case in some flavobacteria (17). This genome provides a suitable genetic resource for study of the transcriptional landscape of microbial biopolymer degradation in the pelagic sea.

### Accession number(s).

This whole-genome shotgun project has been deposited in GenBank under accession number QFDA00000000. The version described here is QFDA01000000.

## References

[1] Lucas J, Wichels A, Teeling H, Chafee M, Scharfe M, Gerdts G 2015 Annual dynamics of North Sea bacterioplankton: seasonal variability superimposes short-term variation. FEMS Microbiol Ecol 91:fiv099. doi:10.1093/femsec/fiv099. 26298013

[2] Bennke CM, Krüger K, Kappelmann L, Huang S, Gobet A, Schüler M, Barbe V, Fuchs BM, Michel G, Teeling H, Amann RI 2016 Polysaccharide utilisation loci of *Bacteroidetes* from two contrasting open ocean sites in the North Atlantic. Environ Microbiol 18:4456–4470. doi:10.1111/1462-2920.13429. 27348854

[3] Seo J-H, Kang I, Yang S-J, Cho J-C 2017 Characterization of spatial distribution of the bacterial community in the South Sea of Korea. PLoS One 12:e0174159. doi:10.1371/journal.pone.0174159. 28306743 PMC5357018

[4] Gómez-Pereira PR, Fuchs BM, Alonso C, Oliver MJ, van Beusekom JEE, Amann R 2010 Distinct flavobacterial communities in contrasting water masses of the North Atlantic Ocean. ISME J 4:472–487. doi:10.1038/ismej.2009.142. 20054356

[5] Yang C, Li Y, Zhou B, Zhou Y, Zheng W, Tian Y, Van Nostrand JD, Wu L, He Z, Zhou J, Zheng T 2015 Illumina sequencing-based analysis of free-living bacterial community dynamics during an *Akashiwo sanguine* bloom in Xiamen sea, China. Sci Rep 5:704. doi:10.1038/srep08476. PMC4329561 25684124

[6] Hattenrath-Lehmann TK, Gobler CJ 2017 Identification of unique microbiomes associated with harmful algal blooms caused by *Alexandrium fundyense* and *Dinophysis acuminata*. Harmful Algae 68:17–30. doi:10.1016/j.hal.2017.07.003. 28962978

[7] Teeling H, Fuchs BM, Bennke CM, Krüger K, Chafee M, Kappelmann L, Reintjes G, Waldmann J, Quast C, Glöckner FO, Lucas J, Wichels A, Gerdts G, Wiltshire KH, Amann RI 2016 Recurring patterns in bacterioplankton dynamics during coastal spring algae blooms. eLife 5:e11888. doi:10.7554/eLife.11888. 27054497 PMC4829426

[8] Gómez-Pereira PR, Schüler M, Fuchs BM, Bennke C, Teeling H, Waldmann J, Richter M, Barbe V, Bataille E, Glöckner FO, Amann R 2012 Genomic content of uncultured *Bacteroidetes* from contrasting oceanic provinces in the North Atlantic Ocean. Environ Microbiol 14:52–66. doi:10.1111/j.1462-2920.2011.02555.x. 21895912

[9] Ngugi DK, Blom J, Alam I, Rashid M, Ba-Alawi W, Zhang G, Hikmawan T, Guan Y, Antunes A, Siam R, El Dorry H, Bajic V, Stingl U 2015 Comparative genomics reveals adaptations of a halotolerant thaumarchaeon in the interfaces of brine pools in the Red Sea. ISME J 9:396–411. doi:10.1038/ismej.2014.137. 25105904 PMC4303633

[10] Bolger AM, Lohse M, Usadel B 2014 Trimmomatic: a flexible trimmer for illumina sequence data. Bioinformatics 30:2114–2120. doi:10.1093/bioinformatics/btu170. 24695404 PMC4103590

[11] Bankevich A, Nurk S, Antipov D, Gurevich AA, Dvorkin M, Kulikov AS, Lesin VM, Nikolenko SI, Pham S, Prjibelski AD, Pyshkin AV, Sirotkin AV, Vyahhi N, Tesler G, Alekseyev MA, Pevzner PA 2012 SPAdes: a new genome assembly algorithm and its applications to single-cell sequencing. J Comput Biol 19:455–477. doi:10.1089/cmb.2012.0021. 22506599 PMC3342519

[12] Parks DH, Imelfort M, Skennerton CT, Hugenholtz P, Tyson GW 2015 CheckM: assessing the quality of microbial genomes recovered from isolates, single cells, and metagenomes. Genome Res 25:1043–1055. doi:10.1101/gr.186072.114. 25977477 PMC4484387

[13] Yin Y, Mao X, Yang J, Chen X, Mao F, Xu Y 2012 dbCAN: a web resource for automated carbohydrate-active enzyme annotation. Nucleic Acids Res 40:W445–W451. doi:10.1093/nar/gks479. 22645317 PMC3394287

[14] Bowers RM, Kyrpides NC, Stepanauskas R, Harmon-Smith M, Doud D, Reddy TBK, Schulz F, Jarett J, Rivers AR, Eloe-Fadrosh EA, Tringe SG, Ivanova NN, Copeland A, Clum A, Becraft ED, Malmstrom RR, Birren B, Podar M, Bork P, Weinstock GM, Garrity GM, Dodsworth JA, Yooseph S, Sutton G, Glöckner FO, Gilbert JA, Nelson WC, Hallam SJ, Jungbluth SP, Ettema TJG, Tighe S, Konstantinidis KT, Liu W-T, Baker BJ, Rattei T, Eisen JA, Hedlund B, McMahon KD, Fierer N, Knight R, Finn R, Cochrane G, Karsch-Mizrachi I, Tyson GW, Rinke C; Genome Standards Consortium, Lapidus A, Meyer F, Yilmaz P, Parks DH, Eren AM, Schriml L, Banfield JF, Hugenholtz P, Woyke T 2017 Minimum information about a single amplified genome (MISAG) and a metagenome-assembled genome (MIMAG) of bacteria and archaea. Nat Biotechnol 35:725–731. doi:10.1038/nbt.3893. 28787424 PMC6436528

[15] Angiuoli SV, Gussman A, Klimke W, Cochrane G, Field D, Garrity G, Kodira CD, Kyrpides N, Madupu R, Markowitz V, Tatusova T, Thomson N, White O 2008 Toward an online repository of standard operating procedures (SOPs) for (meta)genomic annotation. Omics 12:137–141. doi:10.1089/omi.2008.0017. 18416670 PMC3196215

[16] Aziz RK, Bartels D, Best AA, DeJongh M, Disz T, Edwards RA, Formsma K, Gerdes S, Glass EM, Kubal M, Meyer F, Olsen GJ, Olson R, Osterman AL, Overbeek RA, McNeil LK, Paarmann D, Paczian T, Parrello B, Pusch GD, Reich C, Stevens R, Vassieva O, Vonstein V, Wilke A, Zagnitko O 2008 The RAST Server: rapid annotations using subsystems technology. BMC Genomics 9:75. doi:10.1186/1471-2164-9-75. 18261238 PMC2265698

[17] Gómez-Consarnau L, González JM, Coll-Lladó M, Gourdon P, Pascher T, Neutze R, Pedrós-Alió C, Pinhassi J 2007 Light stimulates growth of proteorhodopsin-containing marine flavobacteria. Nature 445:210–213. doi:10.1038/nature05381. 17215843

